# Using Large Languge Models for Processing Sensor Data

**DOI:** 10.3390/s25144380

**Published:** 2025-07-13

**Authors:** Maciej Hojda

**Affiliations:** Faculty of Information and Communication Technology, Wroclaw University of Science and Technology, Wyb. Wyspiańskiego 27, 50-370 Wrocław, Poland; maciej.hojda@pwr.edu.pl

**Keywords:** sensors, data extraction, data reusability, Large Language Models

## Abstract

The wide availability of sensor data stored in multiple formats makes it difficult to reuse in other applications. We consider the problem of extracting sensor data from unstructured and semi-structured texts using Large Language Models. With careful prompt crafting, we have been able to establish a strict JSON structure which can be further processed with automated ease. We establish a workflow that enables the extraction of data using GPT-4, Llama 3, Mistral and Falcon models, and we show that while the closed-source GPT-4 model is generally leading in conversion efficiency, other open-source models can follow this if given appropriate data structures. We define new measures to simplify the comparison, and we present a multi-purpose workflow for sensor data extraction. We observe that some of the smaller models are incapable of correctly extracting data from freeform text but are skilled in processing tabular data. On the other hand, larger models are more robust and avoid conversion mistakes more easily.

## 1. Introduction

Sensor data are available in a multitude of formats from raw numbers obtained directly from the sensor to natural language representations. The type of representation is directly related to the ability of external agents to process, apply and re-use the data. Some formats are easier to process—they are structured in an explicitly organized way such as CSV, JSON or XML files. Conversely, expressing sensor data in natural language inhibits automated processing.

The Internet of Things [1], consisting of billions of devices connected in a network, is a constant generator of sensor data. The heterogeneity, dimensionality and volume of generated information make it difficult to properly process and use it in actual applications of which there are many: green transportation systems [2], weather monitoring systems [3] or medical systems [4] to name a few. This large volume of raw data is, by default, not easily reusable. According to the FAIR Data Principles [5]—FAIR being Findable, Accessible, Interoperable and Reusable—a more robust format for data storage is required. In this paper, we focus on parsing sensor data provided in a variety of formats to a single, uniform structure. We pick a rigid JSON format and use Large Language Models (or LLMs) [6] to perform the conversion. Our conversion consists of extracting the data desired by the user and putting it into a new data structure so that it is more easily processible. We show that the application of a well-generated prompt ensures that the output JSON structure is uniform and ready for further processing.

An LLM is a neural network that takes some text, possibly in natural language, and outputs another text. The parameters of such a network (that is, weights and biases) can range in the billions, which is the reason for the adjective large. LLMs have recently grown in popularity due to their ability to solve various tasks such as translation, giving instructions, creative writing and text analysis. We focus on their ability to extract and transform data provided in a variety of formats to a reusable JSON structure.

## 2. State of the Art

The earliest forays into language models, which LLMs are a specific type of, can be attributed to Shannon [7], who presents one of the first communication prototypes. Traditionally, n-grams were the data structure used as a means of representation. More recently, with the advent of neural networks, their new types such as recurrent neural networks [8], convolutional neural networks [9] or long short-term memory networks [10] paved way to the up and coming transformer networks [11]. Together with the explosion of powerful computational graphic processing units, this led to the emergence of models with a larger number of parameters and great text processing ability. That in turn caused the creation of the GPT-3 model with over 175 billion parameters [12], which is the backbone of the popular ChatGPT service. Other models soon followed [13,14,15]. The scene of LLMs is varied and quickly expanding; for more details on modern techniques such as data annotation and prompt engineering, the reader is refereed to one of the many available surveys [16,17].

Among the publicly available LLMs, the leading position typically falls to GPT-4 and its predecessor GPT-3.5, both of which are employed in ChatGPT. This tool is widely used for data extraction and analysis and is a great benchmark for other language models. It is claimed by the authors of ref. [18] that ChatGPT is a useful tool for performing risk of bias analysis and data extraction from a randomized controlled trial. They further ensure that thanks to the ability to retain context and coherence, ChatGPT can respond to complex queries and be a versatile tool in handling factual inquiries, explanatory responses, content generation and problem-solving assistance. In ref. [19], the authors evaluate ChatGPT for its ability in data race detection in parallel programming. Their conclusion is that with the use of prompt chaining and few-shot prompting, the tool achieves high accuracy in single-bug-finding tasks. On the other hand, using chain-of-thought and few-shot prompting allows ChatGPT to engage in structured reasoning, which performs accurately on multiple-bug-finding tasks. Finally, paper [20] highlights the ability of ChatGPT to extract structured data from clinical notes. The authors develop an LLM-based workflow which utilizes prompt engineering, and it is applied with great success for classification in a pediatric osteosarcoma dataset. In this paper, we use ChatGPT in a twofold way: to evaluate its ability to extract structured data and as a benchmark for other models.

In the papers [21,22], the authors apply LLMs to convert raw sensor data into structured formats. They identify three main challenges for making sensor data interoperable and reusable: the heterogeneous characteristics of sensor data, the unknown anticipated application of sensor data, and variations in the architecture of sensor systems. To alleviate those issues, the authors propose a methodology that employs LLMs in converting semi-structured (such as HTML) sensor data into interoperable formats such as XML and JSON. The results, however, do not enforce a specific output data structure. In our paper, we enforce a specific output data structure which is typically a list of sensing events where each event is described with exactly the same JSON fields. We use careful prompt crafting to ensure the output stays consistent between different prompting rounds. This type of prompt crafting consists of selecting sentences that facilitate the desired output of an LLM.

The authors of ref. [23] use LLMs to solve a joint named entity recognition and relation extraction problem. They convert medical data into a JSON structure. They perform their experiments on three tasks: solid state impurity doping, metal–organic frameworks and materials information extraction. Similarly to our paper, the authors use GPT-3 and Llama 2 for those tasks (we use GPT-4 and Llama 3); however, different to our paper, the authors do not use a rigid JSON structure and apply their methods to medical data rather than sensor data.

It is clear from the literature that the problem of data extraction and conversion with the use of LLMs has already been studied. Extracting is understood here as identifying desired sensor outputs and obtaining their values. In paper [24], the authors study the applicability of LLMs to data extraction from unstructured and semi-structured electronic health records. They process medical notes and perform a series of binary classification tasks to verify the presence of postoperative complications after cardiac surgery. Medical notes were divided into two parts: structured and unstructured. The authors test 19 LLMs and obtain F1 scores varying from 0.992 to 0.408 with GPT-4 scoring very well, reaching 0.979.

Paper [25] contains an evaluation of an LLM’s applicability to the task of tabular data processing. The authors process the WikiTableQuestions dataset comprising 421 tables in order to answer questions about the table contents. The paper considers three types of perturbations: the shuffling of rows, transposition of tables, and both. The authors obtain results with an accuracy of 73.6%, which significantly improves upon the previous work of ref. [26] with 65.9% accuracy. In conclusion, the authors emphasize that although LLMs are sensitive to the structural variance of tables, using a normalization strategy can help mitigate that sensitivity.

The authors of ref. [27] present ChatExtract, which is a method for the automation of materials data extraction from research papers. The presented multi-step workflow enables the extraction of a triplet (Material, Value, Unit) concerning critical cooling rates for metallic glasses. The results for the tested LLMs (GPT-4, GPT-3.5, LLaMA2) are very promising with precision and recall for the GPT-4 model reaching 90.8% and 87.7%. Also present is a generalized workflow which can be used for different data extraction tasks, both single-valued and multi-valued. The authors conclude that approaches such as ChatExtract will grow in usefulness as the capabilities of LLMs grow.

In paper [28], a task of assessing the feasibility of LLMs for data extraction in systematic reviews was presented. In the main tests, the authors assess the ability of an LLM to extract data from abstracts of research reports. The resulting accuracy is 82% for human clinical, 80% for animal and 72% for social science domains. The authors conclude that this is not yet sufficient to consider LLMs as fully automated tools but sufficient to use them as aides that speed up spotting the relevant information for manual reviewers.

Unlike in the literature, in our paper, we consider the following scenario. Sensor data are in a non-interoperable format. The question is: how can we use LLMs to transform the data so that the resulting structure is easily reusable? Therefore, the main goal of the paper is to verify the viability of LLMs in the process of extracting structured data from unstructured or semi-structured sensor data.

We consider three vastly different types of input data. First, we process semantically involved unstructured text where sensor data are embedded in descriptions of individual days or nights. Then, we process data given in mostly tabular form, which is either column oriented or row oriented, where each column or row represents a different sensing event to capture and process. We show that LLMs of varying types can process the data with different degrees of accuracy. The novelty of our approach lies in that we consider long passages of data from which multiple sensing events are extracted. Rather than answering a set of questions or summarizing simple passages, we extract long data structures which are often correlated. There are implicit temporal constraints between subsequent sensing events which makes it harder to extract the data correctly. Using LLMs for this task is, according to our findings, a novel data processing application that deserves separate consideration; such is the reason for writing this paper.

## 3. Materials and Methods

The problem considered in the paper is the conversion of sensor data (and sensor data predictions) to a reusable format which is a given JSON structure. We use LLMs as the conversion tools. The issue is to generate a prompt with enough detail to facilitate a smooth conversion. This requires providing a list of JSON fields within the prompt as well as their ordering and format (data type). It, together with an LLM-specific prompt (if any), gives input which is provided to the LLM.

### 3.1. Datasets

In our experiments, we use weather sensor data (and sensor data predictions) from weather forecasting portals. The portals we chose to use are http://weather.maniac.com and http://www.weather.gov. The first one provides weather data in the natural language; the second one offers a variety of structures.

We consider three separate datasets with distinct qualities. They are abbreviated to Alaska, Area and Temperature. Each dataset consists of ten separate texts. Each text contains multiple sensing events (such as hour, day or location), and each event consists of several sensor readouts that were taken under consideration. Table 1 summarizes the datasets.

#### 3.1.1. Alaska

**Figure 1:**
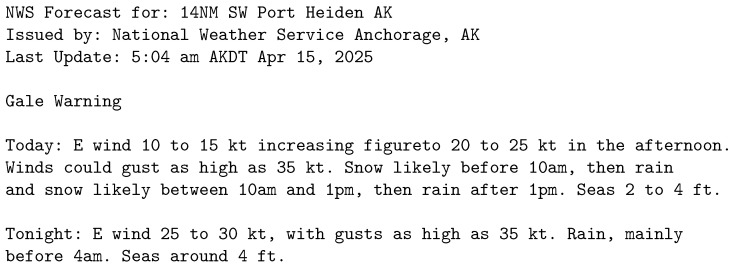
Alaska dataset sample.

The Alaska dataset (Figure 1) consists of pieces of unstructured text which contain weather reports from various weather stations in the Alaska region over the period of 15–21 April 2025. The dataset contains alternating descriptions of days and nights with some reports starting with an overnight instance (which caused poor performance in some of the tested models). The dataset contains wind reports, including direction, speed and possible gust speed. Then, there are temperature reports with low and high temperature mentioned occasionally. Finally, there are predictions of precipitation, cloudiness and snow. The report contains the location and the time of the last update, which is the starting point for the predictions.

#### 3.1.2. Area

**Figure 2:**
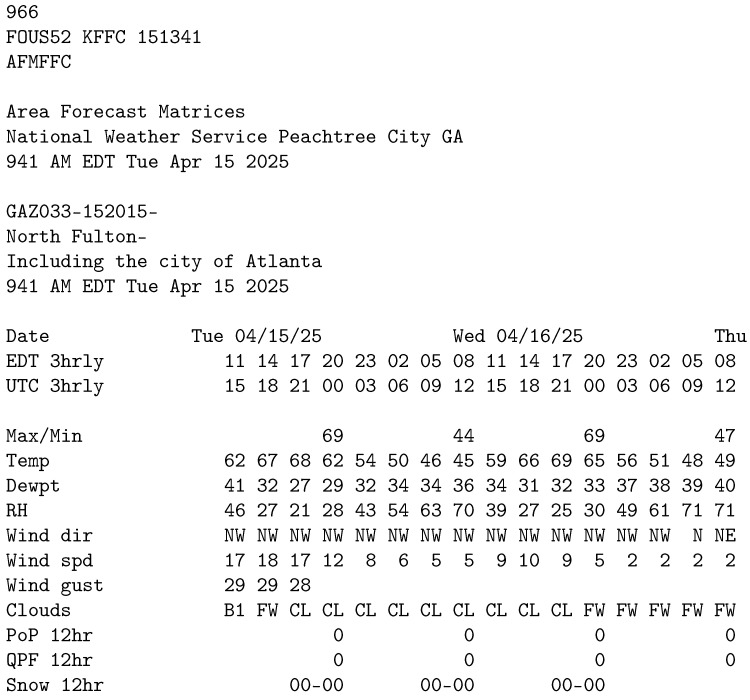
Area dataset sample.

The Area dataset (Figure 2) consists of tabular data embedded within descriptive text fragments. Each instance of data consists of various parameters given in rows and times of day given in columns. The time spans over three days in three-hour intervals while the parameters include temperature, relative humidity, wind direction, wind speed, gust strength, cloud predictions and others. To complicate the layout, time measurements is given in EDT and UTC formats, and not every parameter is filled for every time moment. We trimmed the elements of this dataset to remove unused time moments.    

#### 3.1.3. Temperature

**Figure 3:**
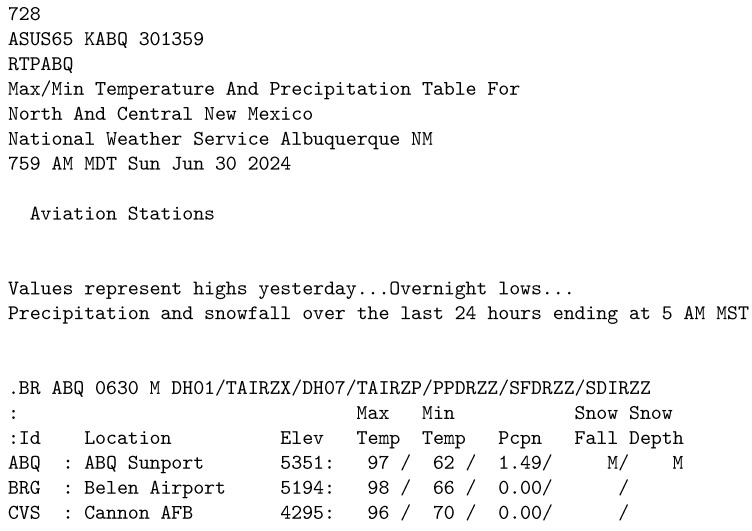
Temperature dataset sample.

The Temperature dataset (Figure 3) consists of a list of locations that is embedded within descriptive text fragments. Each element of the list consists of an abbreviation, location name, elevation, temperatures (minimal and maximal) precipitation and snowfall reports. It is the simplest in layout of all three datasets.

### 3.2. Workflow

We follow a 3-step workflow in order to leverage the benefits of proper prompt design. The workflow is as follows:

1. Data enumeration;
2. Query design;
3. Data retrieval.

**Figure 4:**
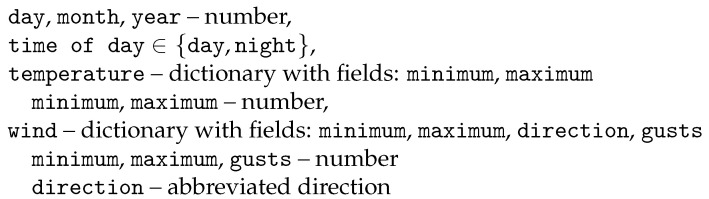
Data enumeration—example fields (Alaska).

In step 1, it is up to the user to decide what kind of fields are to be extracted from a given dataset. The fields can represent either sensor data in the strict sense, such as *temperature*, *wind speed* or *precipitation* either given directly or with modifiers like *maximum temperature* or *average wind speed*. Alternatively, the user can demand fields that naturally co-exist with sensor data and give it meaning, such as *date*, *time of day* or *hour*. With each field, we associate a data type such as *number* or *string* or a more specific expression, such as a *list* of values. It is clear that the fields selected by the user will have a direct impact on the type and the quality of the data retrieved. As an illustrative example, let us assume that the user has selected the fields as in Figure 4.

**Figure 5:**
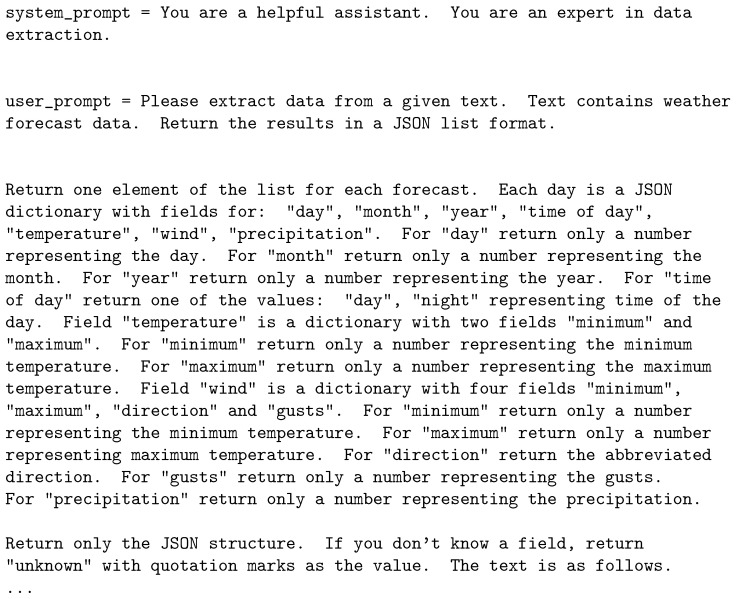
Query design—prompt example (Alaska).

In step 2, we generate a query to facilitate the data retrieval of the fields that were previously selected. The query enables the LLM to return parsed sensor data but is also LLM-specific. Different models have different requirements regarding the structure of the query and the special tokens that have to be used to ensure accurate and useful text generation. We use the Instruct models so our user query will have the sample form as in Figure 5.

As can be seen from the example given, the prompt consists of several parts. The first part, the so-called system prompt, defines the role of the LLM. In this case, since data extraction is the main goal of the task, we also ensure that the prompt includes a statement to reflect that fact. The second part, the user prompt, is crafted to facilitate data extraction by providing the information necessary for the LLM to format the output in a desired way. It is imperative to explain the structure of the output data so as to minimize the mistakes and uncertainty in the workings of the model.

In our case, the user prompt contains three parts. The first part gives the rough idea of the task. It describes the input and the output data to the LLM. It also explains that the output needs to be a JSON list of dictionaries. The second part details the output, providing a list of fields to be included in each dictionary. The basic idea for the second part is to list every field in the structure along with a short explanation of what is supposed to be contained in the field. If the field is a complex structure such as a dictionary, we state that and then we describe all the elements contained within. The third part trims the output so that it contains only the required JSON structure and minimizes hallucinations by permitting the use of unknown as the field value when the content of a particular field cannot be obtained from the input. Finally, the fourth part is the text from which we extract the JSON structure. Additional details on the prompt are available in Appendix A.

**Figure 6:**
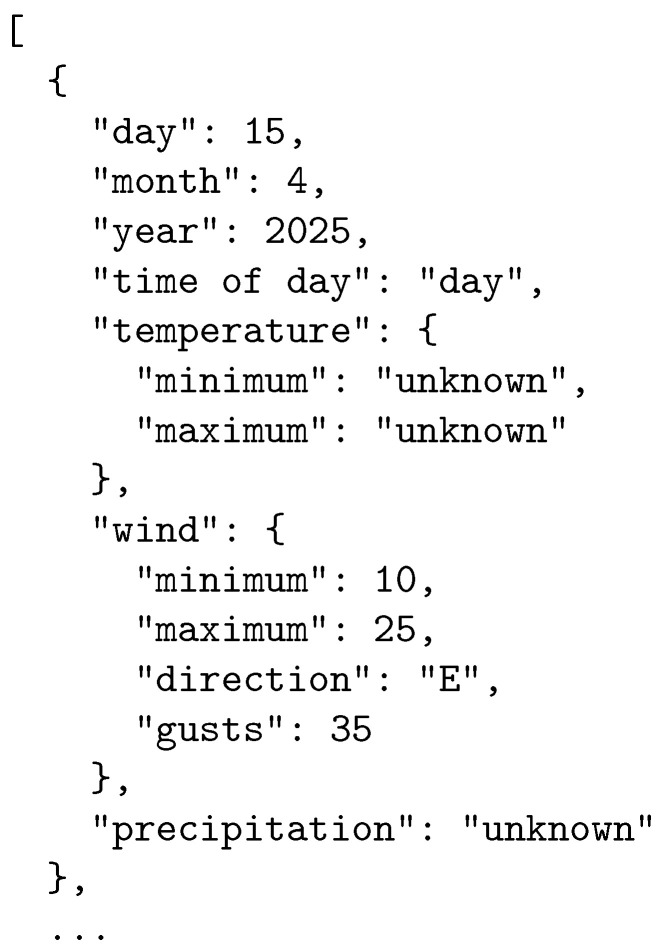
Data retrieval—example structure (Alaska).

In step 3, we feed the query to the LLM at hand, and we obtain the results of data extraction in a structured form. A sample of the data retrieved is presented in Figure 6.

The workflow can be easily generalized and extended into different data retrieval scenarios, where all the three steps are repurposed. It is necessary for the user to generate the prompt based on the requirements of the scenario. Depending on the complexity of the constraints on the data to be retrieved, it might be a work-intensive process. It is also important to note that the more complex the prompt, the larger the context requirements for inference. To sum it up, we also provide a more algorithmic representation of the user prompt generation in Algorithm 1.
**Algorithm 1** Prompt generation algorithmMAIN  1: Define *d* to be the text to extract from.  2: Define *e* to be the type of the text.  3: Define *f* to be the format of results.  4: Define F={1,2,…,F} to be the set of indexes of all fields to be extracted.  5: Define ${\left(a_{i},b_{i},c_{i}\right)}_{i\in F}$ where  6:     $a_{i}$ is the name of the field,  7:     $b_{i}$ is the type of the field (or list of values),  8:     $c_{i}$ is the list of indexes of sub-fields.  9: Define G⊂F to be the subset of indexes of fields that are not sub-fields. 10: Let *P* be a prompt asking to extract data from text of type *e* and format the results as *f*. 11: **For** every i∈G 12:     Let *P* += $\mathrm{EXPAND}(a_{i},b_{i},c_{i})$. 13: Expand *P* by error-correcting statements. 14: Expand *P* by the text *d*. 15: **return** *P*. EXPAND
(a,b,c)  1: **If** *c* is empty  2:     **return** a prompt asking for the field named *a* with type *b*.  3: **Else**  4:     Let *Q* be a prompt asking for the extraction of fields indexed in *c*.  5:     **For** every $s\in c_{i}$  6:        Let *Q* += $\mathrm{EXPAND}(a_{s},b_{s},c_{s})$.  7:    **return** *Q*.


### 3.3. Experiment Design

We conduct experiments using the workflow detailed in the previous sections. The experiments differ in the data that are subject to extraction as well as the prompt formulation. We perform the experiments using five types of LLMs: GPT-4o, Metal Llama 3.1 70b Instruct, Metal Llama 3.1 8b Instruct, Mistral 7b Instruct v0.3 and Falcon3 7b Instruct. They are further abbreviated to GPT, 70b, 8b, M7b and F7b. We run the first model using an online available resource while the Llama models are run locally. Additionally, to facilitate speedy inference, we quantize the locally run models to Q5_K_S. We use both the closed-source GPT and the open-source Llama, Mistral and Falcon. That is due to a fundamental belief that there is an inherent risk carried with closed source solutions—the models can be retracted and the parameters of the network can be changed without warning, leading to usability and reproducibility issues. We do, however, acknowledge the leading position of the GPT model and treat it as both a standalone model for which we evaluate the efficiency and as a baseline for other models.

We performed all local experiments on Debian Bookworm Stable with the 6.1.0-33-amd64 kernel. The programming language used was Python 3.11.2. For all inference, we applied the python-llama-cpp 0.3.3 library (version 0.3.9 was used for the Falcon model due to compatibility issues). The parameters of inference were as follows: temperature = 0.8, top_k = 40, top_p = 0.9, context_length = 4096. Inference was made on an AMD Ryzen Threadripper PRO 5955WX with 256 GB DIMM DDR4 2133 MHz RAM and a triple Radeon RX 7900 XTX. We used ROCM 6.1.2. Experiments with ChatGPT were performed using the chat interface available at http://chatgpt.com.

Due to the random character of LLM-based data extraction, each prompt was fed to the LLMs a total of 3 times. This diminishes the randomness and provides results which are more stable. Each dataset has been manually annotated with correct answers to the given prompts. To evaluate the LLMs, we compare the annotations with the inference output.

Since the output of the LLMs sometimes contains artifacts such as ”’json marking of the beginning of the JSON structure, we clean those up. Furthermore, we manually ensure that all the necessary fields are correctly enclosed in quotation marks (which has been an issue especially for the smaller models). For some models (mostly Mistral), the JSON structure required additional repairs since the model provided incomplete answers such as by marking with ... all the missing parts. Those markings were likewise removed. We have, however, stopped short of corrections which would require us to add text to the answer and consider still invalid entries as missing.

### 3.4. Quality Criteria

There are two datasets to compare: manually annotated source data and multiple instances of parsed destination data. Our text processing task results in a new set of sensing events and sensor readouts—as extracted by the LLM. We have to compare the results (destination data) with the correct set of events and readouts (source data). This comparison is twofold difficult: on one hand, we need to check how many destination instances were extracted correctly; on the other hand, the destination data can contain new, hallucinated measurements. We need measures which comprehensively compare the two datasets and are capable of measuring the number of hallucinations, too. As a consequence, we select the following two measures to evaluate the efficiency of the extraction.

(1) $$M_{1}=\frac{\left|\mathrm{instances}\mathrm{of}\mathrm{source}\mathrm{data}\mathrm{matched}\mathrm{with}\mathrm{destination}\mathrm{data}\right|}{\left|\mathrm{all}\mathrm{instances}\mathrm{in}\mathrm{the}\mathrm{source}\mathrm{data}\right|}$$

(2) $$M_{2}=\frac{\left|\mathrm{instances}\mathrm{of}\mathrm{destination}\mathrm{data}\mathrm{matched}\mathrm{with}\mathrm{source}\mathrm{data}\right|}{\left|\mathrm{all}\mathrm{instances}\mathrm{in}\mathrm{the}\mathrm{destination}\mathrm{data}\right|}$$

Intuitively, the $M_{1}$ is equal to its highest value of 1 when all the instances in the source data were extracted by the model. However, it does not take into account possible hallucinations in the form of extracted instances that are not in the original data. On the other hand, $M_{2}$ has the value of 1 when every instance in the extracted model has its equivalent in the source data. This measure, however, fails to capture the cases when some of the original data are missing in the extracted data.

To alleviate those issues, we introduce a synthetic measure (based on F1)

(3) $$M=\left\{\begin{array}{cc} 2\frac{M_{1}\times M_{2}}{M_{1}+M_{2}} & M_{1}\geq 0,M_{2}\geq 0\\ 0 & M_{1}=0,M_{2}=0 \end{array}\right.$$

that captures the cases when either $M_{1}$ or $M_{2}$ are not equal to 1. Next, we analyze the basic properties of the introduced quality criteria.

**Property** **1.** *If the source data have at least one instance, then $M_{1}$ takes values from [0,1]. It also takes values *0* and *1* for some input.*

**Proof.**  For $M_{1}$, both the numerator and denominator are always positive. Also, since the source data have at least one instance, the denominator is ≥1. The numerator takes the value of 0 when no instances of source data match the destination data, thus resulting in $M_{1}=0$. If all instances of source data match the destination data, then the numerator is equal to the denominator, resulting in $M_{1}=1$. Since you cannot match more instances of source data with destination data than there are instances of source data, that is the largest possible value.    □

**Property** **2.** *If the destination data have at least one instance, then $M_{2}$ takes values from [0,1]. It also takes values *0* and *1* for some input.*

**Proof.**  Analogous to Property 1.    □

**Property** **3.** *If the source data and the destination data both have at least one instance, then M takes values from [0,1]. It also takes values *0* and *1* for some input.*

**Proof.**  For *M*, the numerator takes values from [0,1], while the denominator takes values from [0,2]. Thus, M∈[0,1]. If both $M_{1}=M_{2}=0$, then M=0. When $M_{1}=M_{2}=1$, then M=1.    □

**Figure 7:**
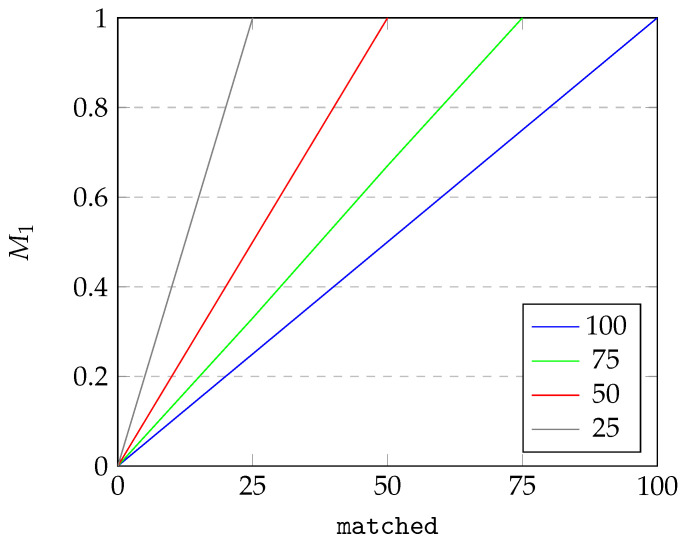
Dependence of $M_{1}$ on matched for varying source.

**Figure 8:**
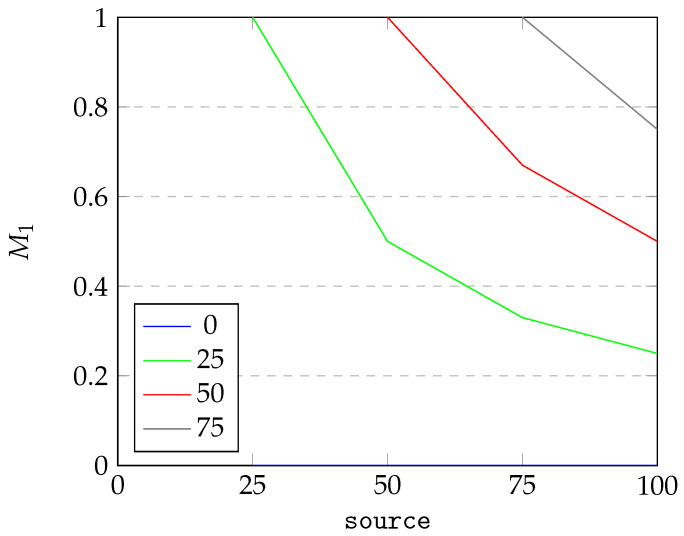
Dependence of $M_{1}$ on source for varying matched.

**Figure 9:**
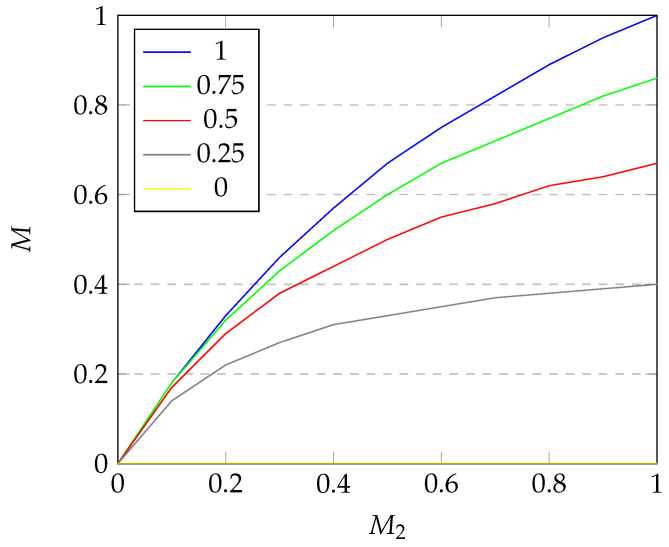
Dependence of *M* on $M_{2}$ for varying $M_{1}$.

Next, we conduct simple sensitivity analysis of the introduced criteria. We perform OAT (one-at-a-time) analysis and visualize the results in Figure 7, Figure 8 and Figure 9.

For Figure 7 and Figure 8, we visualize the dependency of $M_{1}$ on two parameters: the number of instances in the source data (abbreviated source) and instances of source data matched with destination data (abbreviated matched). For the sake of the example, we assume there are at most 100 instances in the source data. As can be seen from Figure 7, under constant source, the quality criterion of $M_{1}$ changes linearly with matched, starting from 0 and reaching 1 when source equates matched. From Figure 8, we can see that the dependence of $M_{1}$ on the source under constant matched is inversly proportional. Starting at 1 for source equating to matched, the value of $M_{1}$ drops until it reaches the minimum possible value. The results for $M_{2}$ are analogous to results for $M_{1}$ and therefore omitted.

For Figure 9, we can see that the dependence of *M* on $M_{2}$ declines as the value of $M_{2}$ rises. The growth of *M* starts elevated and decreases in speed as $M_{2}$ reaches 1. This property is all the more visible for low values of $M_{1}$. The depencence of *M* on $M_{1}$ is analogous and therefore omitted.

Apart from exact matches represented by $M_{1}$, $M_{2}$ and *M*, we separately report the number of sensor readouts that were extracted exactly given correct extraction of the date (*year*, *month*, *day*, *hour*, *time of day*) or location. For this, we introduce a set of analogous measures ${\bar{M}}_{1},\;{\bar{M}}_{2},\;and\;\bar{M}$ (we annotate relevant results with the READOUTS tag). We report two versions of results: either with all values including the unknown fields and without unknown fields (they are abbreviated as ALL and UNK, respectively).

## 4. Results and Discussion

In general, the language models performed the poorest for the Alaska dataset with 8b being virtually useless in the text processing task. The best performance for the Temperature dataset was obtained by the 70b model. For all other experiments, the GPT model took the lead, performing fairly well even on the Alaska dataset. The model performing most poorly was the M7b, which failed to become useful even for the simplest Temperature dataset. Details of the results are reported in the following subsections.

The resulting average inference times are given in Table 2a. Clearly, the larger 70b model had the longest inference times while the smaller 8b, M7b and F7b models performed faster with 8b taking the lead on the Alaska dataset and M7b leading in the Area and Temperature datasets. Additionally, the memory requirements of each model are given in Table 2b.

### 4.1. Alaska

For the Alaska dataset, the performance of the models was the poorest. As can be seen in Table 3, GPT takes the lead with an M-score of 0.779, which is almost twice that of 70b. Despite correctly marking significantly over half of the individual fields, the M-score of 70b stays at 0.4. The main issues for the 70b model stemmed from its inability to correctly identify the first day in many of the series. This caused an off-by-one error which made the entire result infeasible. The results of 8b are negligible—the model managed to correctly identify only 4 instances out of 390 (for the source set) and 385 (for the destination set) in the ALL test. Its scores were slightly better for the UNK test, where it managed to correctly identify 79 instances (for the destination set).

The main issue with M7b was that the model insisted on providing precipitation in textual form instead of a number. This long, descriptive explanation did not conform to requirements, and therefore it made the extraction incorrect. This has a clear impact on the results. Despite performing better on the UNK READOUTS than the F7b model, the *M* measure is worse for M7b. Despite scoring closer on ALL READOUTS to the better F7b model, the ALL non-READOUT result is almost as bad as that of the 8b model. The F7b model had similar issues, and also in several cases, it returned an empty JSON list, suggesting that it did not understand the task outlined before it. However, it still performs much better than the 8b model for both the ALL and the UNK case.

We have decided to evaluate the Alaska dataset for the unquantized 8b model (with f32 parameters) to verify if changing quantization would affect the results in a meaningful way. The results are shown in Table 4. As can be seen, the results are slightly improved, especially in the ALL instances, but the model is still insufficient to provide extraction that could be labeled as acceptable.

### 4.2. Area

In the Area dataset, the performance of each model is significantly increased which is visible in Table 5. Here, GPT again takes the lead, yielding an M-score of 0.983 in both the ALL and the UNK test. Such similarity can be seen in 70b and 8b as well, which yield M-scores of 0.776 and 0.208, respectively. The unsatisfying performance of the 70b model can be attributed to off-by-one errors which happened quite often, resulting in weather parameters being attributed to a period earlier or later than in the source set. Unfortunately, the performance of 8b is still unsatisfactory, although it is not as dismal as in the Alaska dataset. The results highlight the issues the models had with tabular data; however, the performance of all models is significantly better than in the case of fully unstructured text.

For the M7b model, the main issue was that it insisted on shortening the descriptions by adding either ... or otherwise marking the text it considered unneeded. Therefore, the results of the model are poor: almost as bad as in the Alaska dataset. The F7b model simply failed to understand the time given in this tabular form and consistently marked the incorrect hour, sometimes giving hours and minutes despite the latter never appearing in the source text. Unfortunately, those hallucinations gave it *M* scores of 0.002 and 0.003 only.

### 4.3. Temperature

Finally, in the Temperature dataset, four models performed well. As visible in Table 6, surprisingly, the 70b model takes the lead with the correct identification of every single instance and every single field. The M-scores of GPT and 8b are both close to one, being 0.952 and 0.935, respectively (for the ALL test). The models performed better on the source identification task set, showing that they are still capable of hallucinations and can provide data that did not exist in the source text.

Model F7b performed fairly well, reaching *M* scores of 0.764 for the ALL test and an even better 0.834 for the UNK test. Still, it performed worse than the 8b model. The M7b model performed exceedingly poorly. This is due to the fact that it insisted on abbreviating the locations even if explicitly asked not to do so (prompt expanded by Return the location as it is, do not abbreviate it). Since the abbreviations for most of the locations were also incorrect, we have decided to accept this result as a failure of the model in general.

### 4.4. Discussion

Experiments marked as UNK result in better (or equal) overall measures for all tested cases than the ALL experiments for the non-READOUTS case. This suggests that the models are not always capable of deciding when their understanding is limited and thus they are unable to mark as unknown and hallucinate about the measurements. What is surprising, however, is that for the READOUTS experiments, those results do not hold, and it is sometimes the case that despite performing worse for the READOUTS, the non-READOUTS results are better.

Differences between $M_{1}$ and $M_{2}$ measures are inconclusive. Some of the experiments yielded better results for the $M_{1}$ measure, which is the case when the source data were better represented in the extracted destination data. However, the inverse also happens, so sometimes the extracted data are better covered in the destination data; thus, fewer hallucinations were observed. Luckily, the *M* measure gives a joint view of both $M_{1}$ and $M_{2}$ together.

It is important to note that while the GPT performance has been outstanding in most cases, it did not always provide the best results. Such a case has been observed in the Temperature dataset, where a smaller and supposedly less advanced 70b model provided better outcomes. This leads to a belief that in practical cases, using multiple models might provide better overall results than just limiting the conversion process to a single supposed best performer.

We have tested three different types of data. This led to the usage of three different prompts for querying the LLMs. While the performance of the LLMs on different prompts varied, we have noticed that the prompt is susceptible to some variations. For example, not stating explicitly that a field has to contain only one value resulted in some variations to the output. Likewise, without explicitly stating to use question marks around the unknown field, we obtained a lot more unparsable JSON files.

Processed datasets contained text of varying degrees of difficulty for semantic processing. We have noticed that tabular data are more easily transformed than freeform text data. The length of the data does not seem to greatly affect the performance of the LLMs; however, it is important to note that there is a natural limit for the size of data that can be processed, and that is the context limit of the model at hand. This might require additional work such as manually breaking up longer passages of data and modifying queries if the source text is too long.

Finally, 8b, M7b and F7b inference were always significantly faster than 70b inference. This justifies the use of the smaller model if its performance is satisfactory, such as was in the case of the Temperature dataset. On the other hand, if the results are unsatisfactory, for example, if the off-by-one error happens too often, one can improve them by selecting the larger, more capable model.

## 5. Conclusions and Future Work

We show that Large Language Models can be easily applied to the problem of data conversion to interoperable formats such as JSON. The processing of such converted sensor data can be performed automatically with ease, which facilitates reusability. Due to a huge volume of sensor data being generated each second, this conversion process has wide applications in a multitude of data processing systems such as logistics, inspection and monitoring or medicine. We have provided a reusable workflow which uses careful prompt crafting to empower Large Language Models to provide a suitable output, and this workflow can be applied in different contexts also. We have shown that the baseline of the LLM comparison—the ChatGPT employing the GPT-4o model—performs best in freeform text processing, while for more structured text, open source Llama 3 and Falcon models can perform comparably depending on the complexity of the data conversion task. We have noticed, however, that not every LLM is equally capable of processing data. This makes selecting the correct model vital and is strongly dependent on the type of text that is subject to processing.

We find that our results provide a good basis for future research, which can include testing the efficiency of LLMs when converting different data structures. Our workflow can be duplicated in future experiments or compared with other workflows. Furthermore, we make the comparison using self-provided efficiency measures which can also be applied in future expansions of this branch of research.

## Figures and Tables

**Table 1:** Dataset summary.

	Alaska	Area	Temperature
Events	390	600	711
Readouts	2730	3000	2133

**Table 2:** Inference details. (a) Average inference times (in seconds). (b) Tensor buffer size (in MBytes).

**(a)**			
**Model**	**Alaska**	**Area**	**Temperature**
70b	132	151	98
8b	29	41	23
M7b	37	13	13
F7b	34	37	25
**(b)**			
**Model**	**Memory**		
70b	46,395		
8b	5332		
M7b	4770		
F7b	4939		

**Table 3:** Alaska experiment results. (a) Alaska results (ALL). (b) Alaska results (UNK). (c) Alaska results (READOUTS, ALL). (d) Alaska results (READOUTS, UNK).

**(a)**			
**Model**	$M_{1}$	$M_{2}$	M
GPT	0.764	0.795	0.779
70b	0.400	0.400	0.400
8b	0.010	0.010	0.010
M7b	0.028	0.032	0.030
F7b	0.172	0.204	0.187
**(b)**			
**Model**	$M_{1}$	$M_{2}$	M
GPT	0.900	0.808	0.851
70b	0.446	0.431	0.438
8b	0.203	0.010	0.020
M7b	0.167	0.114	0.136
F7b	0.221	0.216	0.218
**(c)**			
**Model**	${\bar{M}}_{1}$	${\bar{M}}_{2}$	$\bar{M}$
GPT	0.905	0.936	0.921
70b	0.677	0.678	0.677
8b	0.238	0.242	0.240
M7b	0.351	0.401	0.374
F7b	0.405	0.481	0.440
**(d)**			
**Model**	${\bar{M}}_{1}$	${\bar{M}}_{2}$	$\bar{M}$
GPT	0.898	0.911	0.905
70b	0.501	0.516	0.508
8b	0.230	0.130	0.166
M7b	0.337	0.331	0.334
F7b	0.291	0.321	0.306

**Table 4:** Alaska experiment results for unquantized 8b. (a) ALL and UNK. (b) READOUTS, ALL and READOUTS, UNK.

**(a)**			
**7b**	$M_{1}$	$M_{2}$	M
ALL	0.054	0.055	0.054
UNK	0.231	0.055	0.088
**(b)**			
**7b**	${\bar{M}}_{1}$	${\bar{M}}_{2}$	$\bar{M}$
ALL	0.265	0.269	0.267
UNK	0.266	0.154	0.195

**Table 5:** Area experiments results. (a) Area results (ALL). (b) Area results (UNK). (c) Area results (READOUTS, ALL). (d) Area results (READOUTS, UNK).

**(a)**			
**Model**	$M_{1}$	$M_{2}$	M
GPT	0.983	0.983	0.983
70b	0.773	0.779	0.776
8b	0.182	0.242	0.208
M7b	0.032	0.127	0.051
F7b	0.002	0.002	0.002
**(b)**			
**Model**	$M_{1}$	$M_{2}$	M
GPT	0.983	0.983	0.983
70b	0.773	0.779	0.776
8b	0.182	0.244	0.208
M7b	0.032	0.140	0.052
F7b	0.002	0.002	0.002
**(c)**			
**Model**	${\bar{M}}_{1}$	${\bar{M}}_{2}$	$\bar{M}$
GPT	0.996	0.996	0.996
70b	0.903	0.909	0.906
8b	0.222	0.301	0.256
M7b	0.041	0.165	0.066
F7b	0.003	0.003	0.003
**(d)**			
**Model**	${\bar{M}}_{1}$	${\bar{M}}_{2}$	$\bar{M}$
GPT	0.996	0.996	0.996
70b	0.903	0.909	0.906
8b	0.222	0.302	0.256
M7b	0.041	0.175	0.067
F7b	0.003	0.003	0.003

**Table 6:** Temperature experiment results. (a) Temperature results (ALL). (b) Temperature results (UNK). (c) Temperature results (READOUTS, ALL). (d) Temperature results (READOUTS, UNK).

**(a)**			
**Model**	$M_{1}$	$M_{2}$	M
GPT	0.952	0.952	0.952
70b	1.000	1.000	1.000
8b	0.935	0.935	0.935
M7b	0.000	0.000	0.000
F7b	0.713	0.823	0.764
**(b)**			
**Model**	$M_{1}$	$M_{2}$	M
GPT	1.000	0.952	0.976
70b	1.000	1.000	1.000
8b	0.996	0.940	0.967
M7b	0.000	0.000	0.000
F7b	0.845	0.823	0.834
**(c)**			
**Model**	${\bar{M}}_{1}$	${\bar{M}}_{2}$	$\bar{M}$
GPT	0.984	0.984	0.984
70b	1.000	1.000	1.000
8b	0.978	0.978	0.978
M7b	0.000	0.000	0.000
F7b	0.815	0.941	0.874
**(d)**			
**Model**	${\bar{M}}_{1}$	${\bar{M}}_{2}$	$\bar{M}$
GPT	1.000	0.983	0.992
70b	1.000	1.000	1.000
8b	0.999	0.979	0.989
M7b	0.000	0.000	0.000
F7b	0.856	0.940	0.896

**Figure A1:**
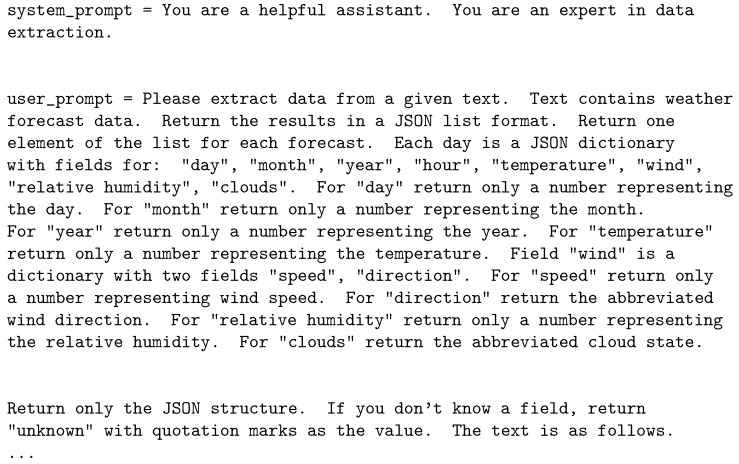
Query design—prompt example (Area).

**Figure A2:**
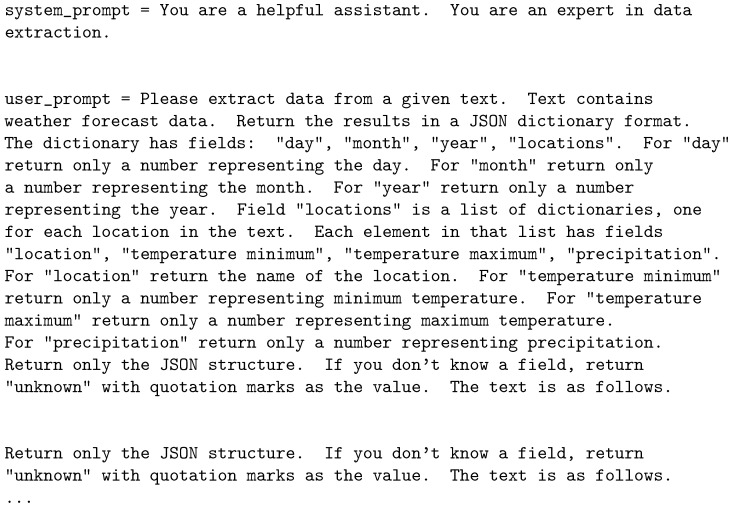
Query design—prompt example (Temperature).

**Figure A3:**
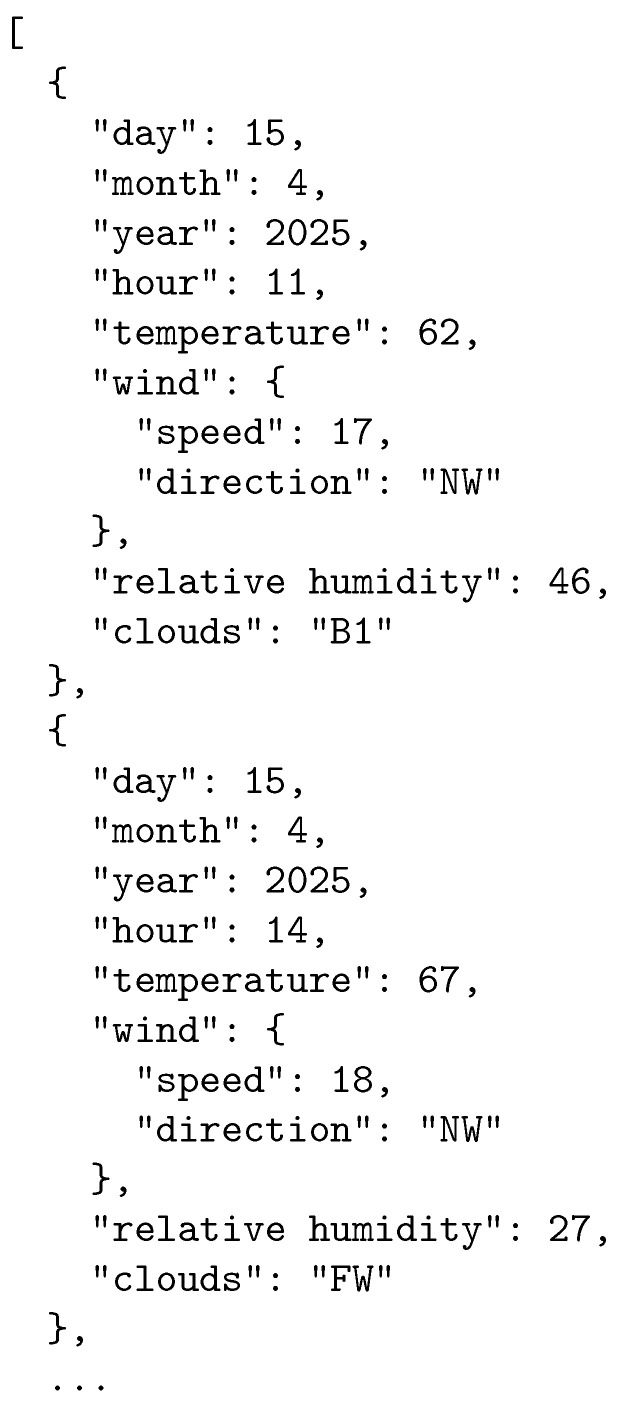
Data retrieval—example structure (Area).

**Figure A4:**
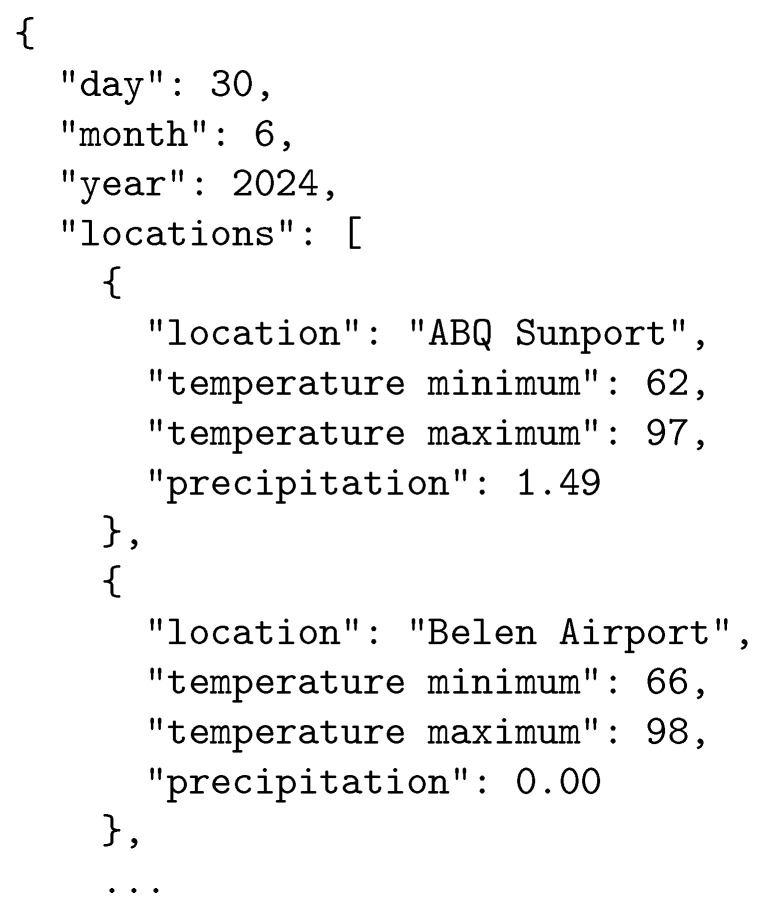
Data retrieval—example structure (Temperature).

**Figure A5:**
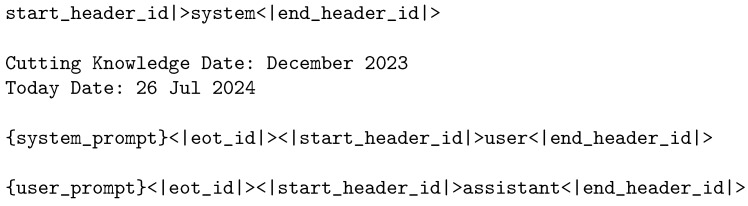
Llama prompt template.

**Figure A6:**
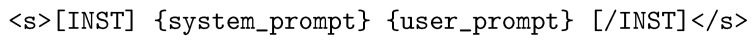
Mistral prompt template.

**Figure A7:**
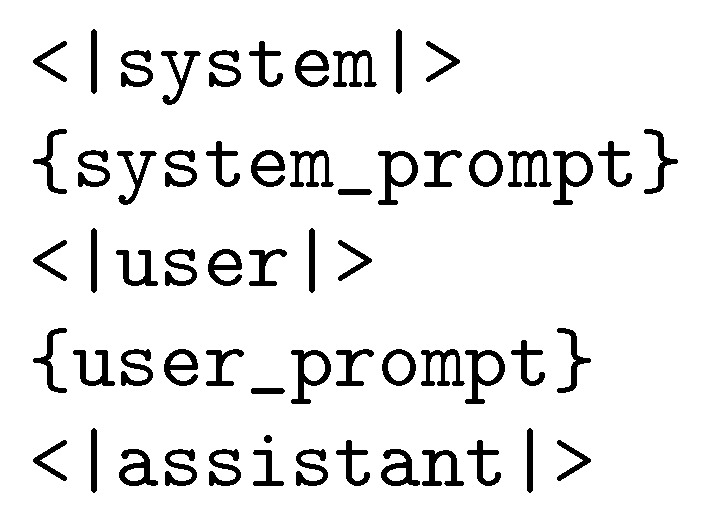
Falcon prompt template.

## Data Availability

Data will be made available to all requestors.

## Appendix A

Here, we include informative examples of prompts for the Area and Temperature dataset as well as partial outputs (Figure A1, Figure A2, Figure A3 and Figure A4).

The prompt comprises two parts—the system and user. Depending on the model, those two parts have to be merged in a different manner. For GPT, the prompt was a direct merge of system and user prompts. Following, we have the prompt template for Llama, Mistral and Falcon models (Figure A5, Figure A6 and Figure A7).
